# Lactic Acidosis Induced by Excessive Use of a Long-Acting Beta Agonist

**DOI:** 10.7759/cureus.63339

**Published:** 2024-06-27

**Authors:** Saho Katsumata, Hirohisa Fujikawa, Takashi Nakamura, Shinko Suzuki

**Affiliations:** 1 Department of Internal Medicine, Suwa Central Hospital, Nagano, JPN; 2 Center for General Medicine Education, School of Medicine, Keio University, Tokyo, JPN

**Keywords:** long-acting beta agonist (laba), rare side effect, aco: asthma-copd overlap, drug/alcohol overdose, lactic acidosis

## Abstract

Lactic acidosis is associated with poorer clinical outcomes in critical care. The causes of this condition are divided into two groups: type A (tissue hypoxia) and type B (metabolic abnormalities). Of these, drug-induced lactic acidosis is categorized as type B and is often overlooked due to clinicians’ poor awareness. We herein report a rare case of drug-induced lactic acidosis due to excessive use of a long-acting beta agonist (LABA) in a patient with asthma-chronic obstructive pulmonary disease overlap exacerbation. He initially presented with markedly elevated lactate and metabolic acidosis with unknown etiology. A detailed medical interview revealed that he had inhaled a large amount of LABA on the day of admission, which led to our final diagnosis. The patient’s respiratory status and lactate levels gradually improved with the appropriate use of inhalation therapy. While there have been many recent reports of lactic acidosis caused by short-acting beta agonists, our case suggests that excessive use of LABAs may also lead to lactic acidosis. Clinicians should be aware of the possibility that LABAs can cause lactic acidosis because poor awareness of the condition may lead to inappropriate patient care.

## Introduction

Lactic acid is a crucial intermediate in the metabolism of carbohydrates and non-essential amino acids [1]. When lactic acid production exceeds its clearance, it accumulates, leading to lactic acidosis [2]. Increased levels of blood lactate are associated with significant morbidity and mortality among patients in critical care settings [3]. Accordingly, prompt intervention for lactic acidosis is usually needed.

According to the Cohen and Woods classification, the causes of lactic acidosis are divided into two categories: type A and type B [4]. Type A is associated with clinically evident hypoperfusion or hypoxia, and type B occurs in patients with metabolic abnormalities. Type A is the most common type, and typical examples include severe hypoxia and shock. Drug-induced lactic acidosis is classified as type B and should always be included in the differential diagnosis of patients taking medications that are at risk of lactic acidosis. However, because it remains a rare cause of lactic acidosis and is not well-known to clinicians, drug-induced lactic acidosis is frequently overlooked.

Herein, we report a case of lactic acidosis induced by a long-acting beta agonist (LABA). The findings of this report will enhance awareness of the condition among clinicians.

## Case presentation

A 74-year-old man with asthma-chronic obstructive pulmonary disease overlap (ACO) presented to our hospital with a one-week history of cough with green sputum and increased shortness of breath. His medical history included dyslipidemia, gastroesophageal reflux disease, and hypertension. He was prescribed budesonide/glycopyrrolate/formoterol fumarate triple therapy (inhaled corticosteroid/long-acting muscarinic antagonist/long-acting beta agonist (ICS/LAMA/LABA)), montelukast, atorvastatin, lansoprazole, and candesartan.

Examination revealed tachypnea of 50 breaths per min, decreased oxygen saturation of 97% on an oxygen mask at 5 L/min, tachycardia of 155 beats per min, and an increased blood pressure of 188/102 mmHg. The patient did not have a fever. Chest examination revealed polyphonic wheezes in both lungs, but he did not have signs of fluid overload. The patient had bilateral hand tremors. Arterial blood gas analysis showed metabolic acidosis (pH 7.306, bicarbonate 14.4 mEq/L) and an increased blood lactate level (11.63 (normal range 0.8-1.77) mmol/L). Chest radiography revealed no evidence of pneumonia or any other acute pathologies. Electrocardiography revealed atrial fibrillation that had never been diagnosed, whereas transthoracic echocardiography showed no remarkable findings. Blood, urine, and sputum cultures did not reveal any pathogens. Initially, the cause of the deteriorating respiratory status of the patient was diagnosed as ACO exacerbation, and therapy with inhalation of a short-acting beta agonist (SABA) (2.5 mg of salbutamol every 6 h), systemic corticosteroids (80 mg of methylprednisolone every 24 h), and antibiotics (2 g of ceftriaxone every 24 h) was initiated. Conversely, because the cause of the lactic acidosis was not determined, the patient was admitted to the intensive care unit for close observation.

**Figure 1:**
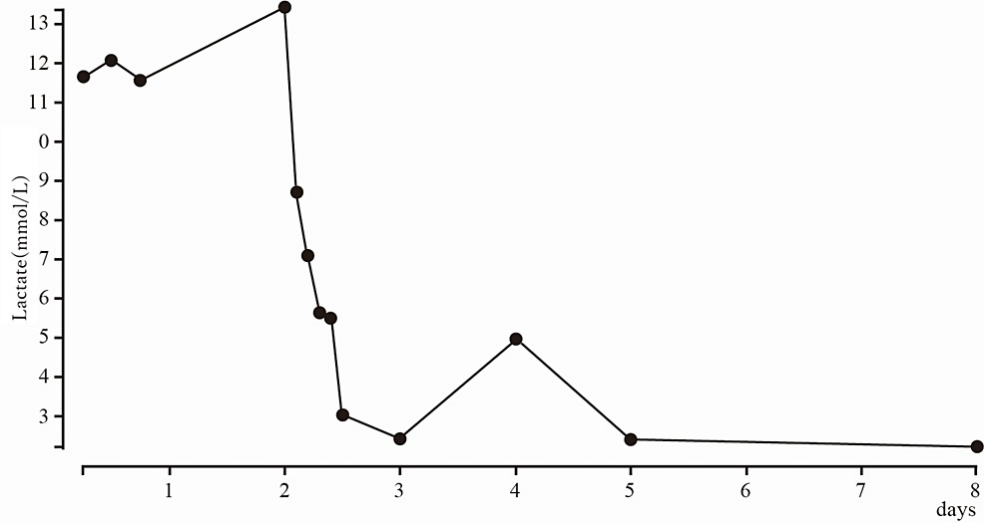
Serum lactate levels

After admission, the patient’s respiratory status and lactate levels gradually improved (Figure 1). We considered various causes of lactic acidosis and eliminated all of them (Table 1). Because additional history-taking revealed that he had inhaled ICS/LAMA/LABA more than 30 times on the day of admission, we diagnosed him with lactic acidosis due to excessive LABA use. The patient was discharged with no complications on the 10th day of hospitalization, according to the instructions for the inhalation technique.

**Table 1:** Causes of lactic acidosis and reasons for exclusion

Type of lactic acidosis	Differential diagnosis	Reasons for exclusion
A	Carbon monoxide poisoning	Normal levels of carbon monoxide hemoglobin: 1.3%.
	Decreased tissue perfusion (local hypoperfusion)	No physical and computed tomography findings suggestive of hypoperfusion (e.g. enteric ischemia).
	Increased glycolysis (i.e., excessive muscular activity, exercise, and trembling)	Absence of excessive muscular activity. No exercise. No trembling observed.
	Seizures	No seizures observed.
	Severe anemia (hemoglobin level <50 g/L)	Normal level of hemoglobin: 152 g/L.
	Severe hypoxia (PaO_2_ <30 mmHg)	The pretreatment degree of hypoxia was not severe: PaO_2_ 95.9 mmHg.
	Shock (systemic hypoperfusion)	No signs of shock (e.g., vital signs, physical examinations, and transthoracic echocardiogram).
B	Sepsis	Negative results for blood, sputum, and urine cultures. Negative result for SARS-CoV-2.
	Inhaled corticosteroid and long-acting muscarinic antagonist	No cases of lactic acidosis have been reported with these medications. The mechanism of actions of these medications makes them very unlikely to cause lactic acidosis.
	Other medications (i.e., atorvastatin, candesartan, lansoprazole, and montelukast)	No cases of lactic acidosis have been reported with these medications. The mechanism of actions of these medications makes them very unlikely to cause lactic acidosis.
	Associated with underlying disease (i.e., diabetic ketoacidosis, leukemia/lymphoma, pheochromocytoma, severe liver disease, thiamine deficiency)	Absence of diabetes. No evidence of leukemia on laboratory examinations. No evidence of lymphoma on laboratory examinations and chest-abdomen-pelvis computed tomography. No history of suspected pheochromocytoma. Normal liver function. Normal level of thiamine: 47.9 mcg/L.
	Associated with congenital metabolic disorders (i.e., mitochondrial myopathy and pyruvate dehydrogenase deficiency)	No clinical symptoms and history suggestive of these congenital disorders.

## Discussion

Lactic acid is a crucial intermediate in the metabolism of carbohydrates and non-essential amino acids [1]. When lactic acid production exceeds its clearance, it accumulates, leading to lactic acidosis [2]. Increased levels of blood lactate are associated with significant morbidity and mortality among patients in critical care settings [3]. Accordingly, prompt intervention for lactic acidosis is usually needed.

According to the Cohen and Woods classification, the causes of lactic acidosis are divided into two categories: type A and type B [4]. Type A is associated with clinically evident hypoperfusion or hypoxia, and type B occurs in patients with metabolic abnormalities. Type A is the most common type, and typical examples include severe hypoxia and shock. Drug-induced lactic acidosis is classified as type B and should always be included in the differential diagnosis of patients taking medications that are at risk of lactic acidosis. However, because it remains a rare cause of lactic acidosis and is not well-known to clinicians, drug-induced lactic acidosis is frequently overlooked.

Drug-induced lactic acidosis is often associated with the administration of metformin, isoniazid, linezolid, propofol, and nucleoside reverse transcriptase inhibitors. Metformin-associated lactic acidosis (MALA) is the most common among these conditions. Metformin is a first-line oral therapy with multifaceted effects in type 2 diabetes. However, MALA is a serious condition that occurs in patients with risk factors and has a high mortality rate [5].

Recently, lactic acidosis induced by SABAs has attracted attention, and many cases have been reported. Their pathophysiology is as follows: stimulation of beta-adrenergic receptors leads to various metabolic effects, including overproduction of glucose and increased conversion of pyruvate to lactate. Moreover, exaggeration of lipolysis inhibits the conversion of pyruvate to acetyl-coenzyme A with a consequent increase in lactate [6]. A recent systematic review found that 30% of patients with asthma treated with beta-2 agonists developed hyperlactatemia and that there was an association between lactate levels and beta-2 agonist doses [7]. Thus, SABA-induced lactic acidosis is considered more common than clinicians expect and should be treated with caution.

Conversely, there are very few cases of lactic acidosis caused by LABAs (Table 2) [8-10]. All previously reported cases were associated with LABA overuse. LABAs remain on beta-adrenoceptors for an extended period due to their lipophilic profile and high affinity for beta-adrenoceptors [11]. In addition, their duration of action is considered dose-dependent [12]. In this case, a beta-adrenergic state, such as new-onset atrial fibrillation and bilateral hand tremors, was observed, but it steadily resolved with the appropriate use of inhalation therapy. This clinical course suggests that lactic acidosis is mainly caused by a hypermetabolic state resulting from the administration of large doses of LABAs.

**Table 2:** Previous cases of lactic acidosis secondary to excessive long-acting beta agonists

Case no. [Ref]	Age	Sex	Medication	Dose (µg)	Peak lactate level (mmol/L)
1 [8]	83	Female	Salmeterol	3000	4.2
2 [9]	16	Female	Salmeterol/fluticasone	1500/3000	8.3
3 [10]	44	Female	Salmeterol and Salbutamol	Not reported	5.2

In general, LABAs are considered unlikely to cause severe adverse effects when prescribed to patients with stable asthma and/or chronic obstructive pulmonary disease. As noted earlier, very few cases of lactic acidosis related to inhaled beta agonist have been caused by LABAs, whereas many cases of SABA-induced lactic acidosis have been reported. Therefore, in daily clinical practice, it may be difficult to suspect an LABA as a causative agent of lactic acidosis. There is a concern that elevated lactate levels or hyperventilation at the expense of acidosis itself may be misunderstood as inadequate control of the condition, leading to escalating doses of beta-stimulants and further worsening lactic acidosis. As indicated by Table 2, all previous cases of lactic acidosis due to LABAs have been caused by salmeterol, but this case was due to formoterol. Symbicort®, which contains formoterol, is an LABA but is approved for use as a SABA in Symbicort® Maintenance and Reliever Therapy. Currently, formoterol is an ingredient of commonly used inhalants such as Flutiform® and Breztri® Aerosphere®. As these become more prevalent, we may see more cases like the present case. Clinicians should be aware of the possibility that LABAs cause lactic acidosis.

## Conclusions

We encountered an uncommon case of lactic acidosis induced by the overuse of LABAs. A detailed medical interview led to the correct diagnosis. The patient’s condition improved with the appropriate use of inhalation therapy. Our case is insightful because it suggests that LABAs can cause lactic acidosis. Physicians should be aware of this condition because misdiagnosis will lead to inappropriate patient care.
